# A Comprehensive Review on the Behaviour of Motorcyclists: Motivations, Issues, Challenges, Substantial Analysis and Recommendations

**DOI:** 10.3390/ijerph19063552

**Published:** 2022-03-17

**Authors:** Sarah Najm Abdulwahid, Moamin A. Mahmoud, Bilal Bahaa Zaidan, Abdullah Hussein Alamoodi, Salem Garfan, Mohammed Talal, Aws Alaa Zaidan

**Affiliations:** 1 College of Graduate Studies, Universiti Tenaga Nasional, Kajang 43000, Malaysia; sarah_njm@yahoo.com; 2 Institute of Informatics and Computing in Energy, Universiti Tenaga Nasional, Kajang 43000, Malaysia; 3 Future Technology Research Center, National Yunlin University of Science and Technology, Douliu 64002, Taiwan; 4 Department of Computing, Universiti Pendidikan Sultan Idris, Tanjong Malim 35900, Malaysia; alamoodi@fskik.upsi.edu.my (A.H.A.); salem.garfan@gmail.com (S.G.); aws.alaa@fskik.upsi.edu.my (A.A.Z.); 5 Department of Electronic Engineering, Faculty of Electrical and Electronic Engineering, Universiti Tun Hussein Onn Malaysia (UTHM), Batu Pahat 86400, Malaysia; moha_talal2000@yahoo.com

**Keywords:** intelligent transportation system, driver behaviour, traffic violation, motorcyclists

## Abstract

With the continuous emergence of new technologies and the adaptation of smart systems in transportation, motorcyclist driving behaviour plays an important role in the transition towards intelligent transportation systems (ITS). Studying motorcyclist driving behaviour requires accurate models with accurate and complete datasets for better road safety and traffic management. As accuracy is needed in modelling, motorcyclist driving behaviour analyses can be performed using sensors that collect driving behaviour characteristics during real-time experiments. This review article systematically investigates the literature on motorcyclist driving behaviour to present many findings related to the issues, problems, challenges, and research gaps that have existed over the last 10 years (2011–2021). A number of digital databases (i.e., IEEE Xplore^®^, ScienceDirect, Scopus, and Web of Science) were searched and explored to collect reliable peer-reviewed articles. Out of the 2214 collected articles, only 174 articles formed the final set of articles used in the analysis of the motorcyclist research area. The filtration process consisted of two stages that were implemented on the collected articles. Inclusion criteria were the core of the first stage of the filtration process keeping articles only if they were a study or review written in English or were articles that mainly incorporated the driving style of motorcyclists. The second phase of the filtration process is based on more rules for article inclusion. The criteria of inclusion for the second phase of filtration examined the deployment of motorcyclist driver behaviour characterisation procedures using a real-time-based data acquisition system (DAS) or a questionnaire. The final number of articles was divided into three main groups: reviews (7/174), experimental studies (41/174), and social studies-based articles (126/174). This taxonomy of the literature was developed to group the literature into articles with similar types of experimental conditions. Recommendation topics are also presented to enable and enhance the pace of the development in this research area. Research gaps are presented by implementing a substantial analysis of the previously proposed methodologies. The analysis mainly identified the gaps in the development of data acquisition systems, model accuracy, and data types incorporated in the proposed models. Finally, research directions towards ITS are provided by exploring key topics necessary in the advancement of this research area.

## 1. Introduction

By exploring the literature on driver behaviour research, different research dimensions may be found. Over the past years, motorcyclists have constituted major concerns from the road safety perspective [1]. As the wide range of technologies used in our daily lives grows, so does the rate at which they are employed in the transportation sector, and understanding driver behaviour considered an important step towards achieving intelligent transportation systems (ITS) and ensuring road safety [2]. The increasing number of traffic accidents is a serious challenge in many countries. Accidents can harm individuals and governments, particularly regarding social and economic loss, which generally affects society [3]. Globally, traffic road accidents cause more than a million mortalities yearly and are predicted to be the fifth-highest cause of death globally by the year 2030 if no preventive measures are taken [4]. Improving traffic safety is one of the top priorities in many countries [5]. Understanding mixed traffic characteristics is a result of studying motorcycle driver behaviour [6]. Furthermore, understanding the main causes of accidents will help find solutions to prevent or reduce traffic accidents. Behaviour is considered the most critical factor affecting road safety. Driver behaviour can be defined as concepts related to a driver’s driving mannerisms and actions [7]. Thus, analysing and understanding driver behaviour is the key factor in preventing or reducing traffic accidents [3].

Surveying and exploring the research area of motorcyclist driving behaviour revealed a gap in articles that discuss, examine, and analyse the driving behaviour of motorcyclists. This systematic review article presents an exploration and examination of the research area from different aspects, such as the type of source of the dataset used to model driver behaviour, the methodologies used to replicate driving styles, an examination of the incorporation of human-based factors in modelling, and motivational and recommended topics are presented to further increase the development in this research area. Future research directions are provided to accelerate the development in this research area. Accordingly, this study mainly aims to review previous research, summarise their findings related to important requirements for identifying motorcyclist driving behaviour, and determine the methods, estimate techniques, and suggest taxonomic literature. This study was directed towards experimental-based efforts rather than human descriptions or social experience/opinion. This review article is trying to answer the following questions:

1. What are the methods used for data collection on motorcyclist driving behaviour?
2. What are the types of research and methods used to collect real-time-based datasets?
3. What are the methods used for motorcycle driving style analysis and identification?
4. Have age, sample size, and gender been considered in real-time-based experiments?
5. What is the type of methodology used to classify motorcyclist driving styles?
6. What are the features that were adopted in previous academic research for motorcycle driving behaviour recognition?
7. What are the problems in the datasets used for motorcyclist driving behaviour?

The remaining parts of this paper are organised as follows: Section 1 provides the study’s introduction, and Section 2 presents a systematic review protocol description. Section 3 presents a taxonomy, and Section 4 shows the discussion, recommendations, challenges and evaluation methods. Section 5 presents the methodological aspects of previous research, Section 6 presents a substantial analysis, Section 7 presents the comparison between this systematic review article and a previous review article. Section 8 presents the future research directions, Section 9 presents limitations, and lastly, Section 10 concludes the study.

## 2. Systematic Review Protocol

**Figure 1:**
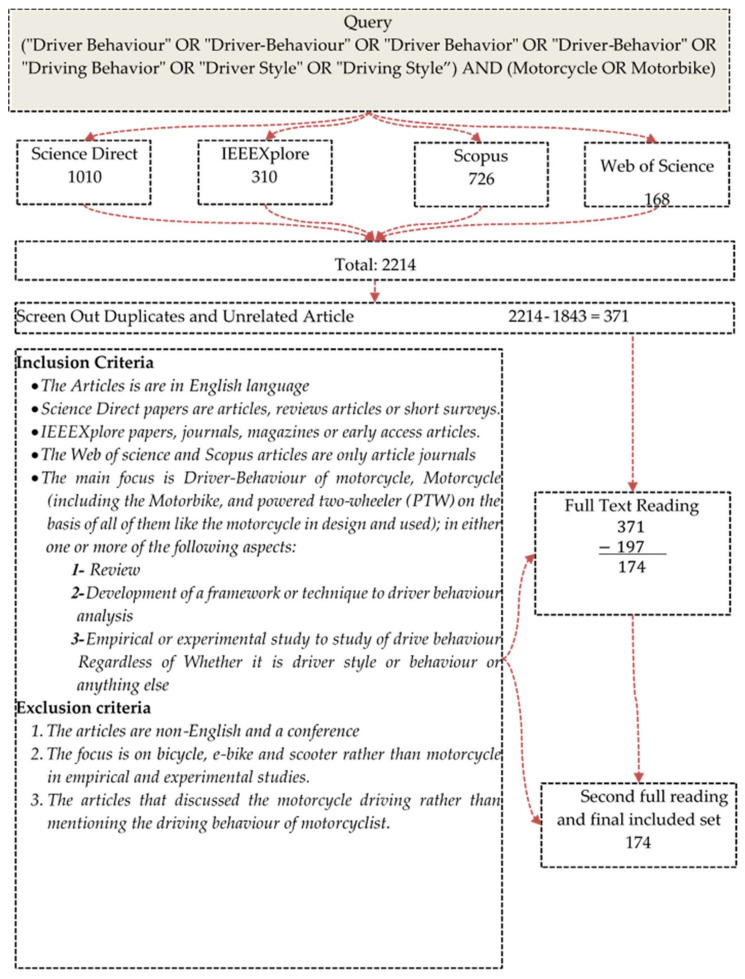
Selection of studies, search queries, inclusion and exclusion criteria.

This section presents a systematic literature review (SLR) protocol. The SLR promotes complete knowledge related to a specific phenomenon or topic of interest and provides significant insights and specifics for future research and policies [8]. The search strategy followed the Preferred Reporting Items for Systematic Reviews and Meta-Analyses (PRISMA) methodology [9], as shown in Figure 1. Four digital databases have been chosen: (1) Science Direct (SD) offers access to journals from various domains, including technical, scientific, and medical journals; (2) IEEEXplore library provides different engineering and technology-based journals; (3) the Scopus database provides different health science, social science, life science, and physical science journal articles; and (4) Web of Science (WoS) contains journals from the humanities, arts, and social sciences. These databases were chosen because of their academic reliability. The search query was updated in July 2021. The upper part of Figure 1 shows the search query. Advanced search options were utilised for selecting papers in each of the search engines, excluding conference papers, book chapters, and other document types. Table 1 presents the settings utilised for applying the search queries.

### 2.1. Selection of Study

**Figure 2:**
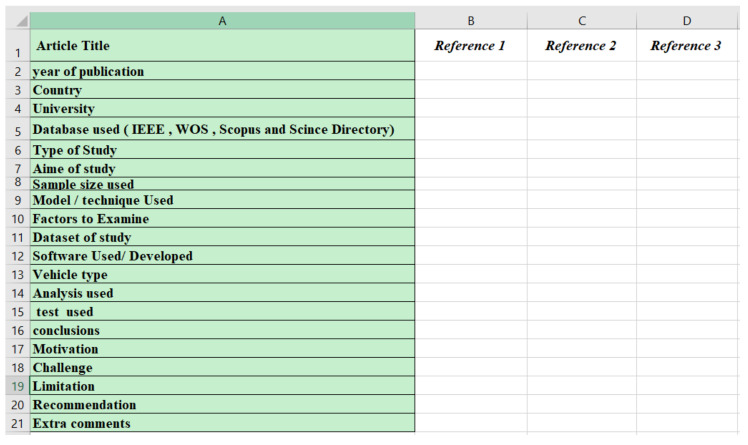
Scanned attributes from full-text reading.

This phase starts with the initial search results of 2214 articles, followed by three consecutive phases. The phases are screening, filtering, and initiating categories for determining if the selected articles might be suitable for the review. Screening is the reviewing process regarding titles and abstracts of articles, while filtering is the process of assessing the eligibility of articles for inclusion by implementing full-text reading. Moreover, duplicated articles were removed from the results. In addition to title scanning, abstract screening was also used to determine the relevant papers, categorised in various folders. The filtering phase commenced by carrying out complete full-text readings of the screened articles from the first phase. Each of the articles was separately analysed, and different attributes were recorded in an Excel sheet (Figure 2). The same procedure was applied to all selected articles.

### 2.2. Eligibility Criteria

During the initial screening process, a set of criteria were required to select the research articles. Any article that satisfied the criteria listed in Figure 1 was selected. An initial target was set to map the research on motorcycle driver behaviour and the general taxonomy of three categories was obtained from the pre-survey literature. Following the duplicates’ initial removal, articles not fulfilling the eligibility criteria were excluded by two operations; namely, filtering and screening. The inclusion criteria are as follows: (1) the articles are in the English language; (2) Science Direct papers are articles, reviews articles, or short surveys; (3) IEEEXplore papers are journal articles, magazines, or early access articles; and (4) the Web of Science and Scopus articles are only article journals. Additionally, the main focus of the articles is the behaviour of motorcycle drivers (including drivers of motorbikes and powered two-wheelers (PTW) on the basis that is a vehicle like the motorcycle in design and use) in one or more of the following aspects: (1) a review article, (2) the development of a framework or technique for driver behaviour analysis, or (3) an empirical or experimental study of driver behaviour, regardless of whether it is driver style, behaviour, or anything else. Furthermore, the exclusion criteria used are the following: (1) the articles are non-English or a conference proceeding; (2) the focus is on bicycles, e-bikes, or scooters rather than motorcycles in empirical and experimental studies; and (3) the articles discuss motorcycle driving rather than mentioning the driving behaviour of motorcyclists.

## 3. Taxonomy

**Figure 3:**
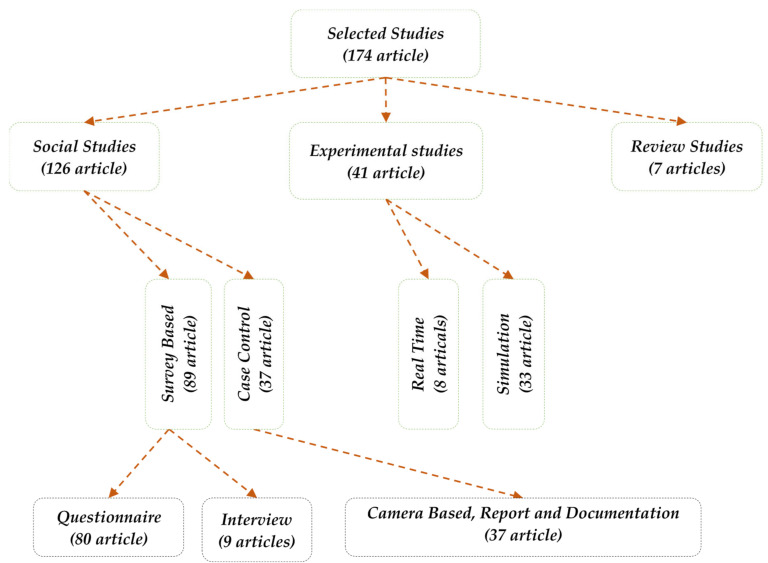
Taxonomy of research literature on motorcycle drivers’ behaviour.

The presented section provides a taxonomy that summarises the search process’s results. This process was initiated after searching, scanning, filtering, and reading the full texts of the selected articles. All the articles are categorised into four main categories and different subcategories. The first category is social science studies (surveys based on questionnaires and case-control studies). The second category is experimental studies which are linked to the factor of motorcycle driver behaviour. This category has two subcategories (real-time field tests and experiments by use of simulations). The third category is the development of apps and systems, with two subcategories (smartphone app development and systems). The final category is reviews, consisting of surveys and review articles. The latter summarises the present state-of-the-art literature on motorcyclists’ driving behaviour. The main categories and their subcategories have been described in the next subsections (Figure 3) and illustrate the taxonomy of research literature on motorcycle drivers’ behaviour.

### 3.1. Social Science Studies

The first main category of taxonomy refers to a kind of study involving data collection including surveys and observations (*n* = 126/174). This section is divided into two subcategories: surveys based on questionnaire techniques and case-control studies.

#### 3.1.1. Surveys Based on Questionnaire and Interview Techniques

The first subcategory of social science strategy studies, namely, surveys based on questionnaire techniques, is related to studies using datasets or data collection methods (*n* = 89/126 articles). Many articles used questionnaire designs (*n* = 80/89 articles), as shown in Table 2.

However, a few of them used a dataset obtained by specifying the associated features and analysing research issues based on statistical approaches. Other studies focused on interview methods (*n* = 9/89 articles), where interviews were held with the aid of a questionnaire, administered using a probabilistic sample [58,74,75,76] online to collect a dataset for analysis [25]. a one-on-one interview survey [41] or face-to-face interviews [43]. An online survey was first designed using Google Forms [22]. In this subgroup, the last article described telephone interviews [42] to identify the context and their shortcomings during the reported events.

It can be pointed out from Table 2 that most authors used driver-behaviour questionnaires to examine the driving style of motorcyclists (14/80). This was followed by the use of self-reported questionnaires (12/80), motorcycle rider behaviour questionnaires (11/80), demographic behaviour questionnaires (8/80), self-administrated questionnaires (8/80), (Buss and Perry) aggressive questionnaires (5/80), standard questionnaires (3/80), the Drivers Angry Thoughts Questionnaire (DATQ) (2/80), the Theory of Planned Behaviour (TPB) questionnaire (2/80), and the Likert questionnaire (2/80). Other questionnaires were only used once each in the surveyed articles.

The comprehensive analysis in Table 2 illustrates that the questionnaire used most was the DBQ rather than other types of questionnaires. Each article was designed to examine special attributes of characteristics according to the needs of their authors. There is no agreement on the number of factors and characteristics that represent motorcyclist driving behaviour, and some authors prefer to include some attributes and neglect others. On the other hand, the authors of some articles used more than one questionnaire to examine the driving style of motorcyclists. This indicates there is no agreement among researchers on the time and number of conditions that can represent the driving style of motorcyclists, leaving a gap for more research to include more factors and characteristics in a single standard motorcycle driver behaviour questionnaire. Moreover, some of the attributes featured are subjective values and can be biased. The question of how to quantify the subjective aspects of motorcyclist driving behaviour is still not answered. Furthermore, the number of respondents varied from one questionnaire to another. This means that there is no clear standardisation to be followed by researchers to produce the most accurate and well-represented dataset.

#### 3.1.2. Case-Control Study

This section involves articles related to the best use of observations to determine the severity of traffic crashes, hazards, road accident consequences, and riding behaviour. Other articles focused on using a camera to observe and record factors that cause motorcycle accidents and determine motorcycle driver behaviours [6,77,78,79,80,81,82,83]. One study focused on the use of MetroCount MC-5600 data loggers for the collection of data associated with the driving of vehicles far from or near minor roads [84]. Another article [85] used aggregate data involving the value dimensions of Schwartz, the law enforcement regarding five risk factors concerning road safety, and the gross national income for each capita in addition to fatality rates in traffic for 97 nations. Moreover, other studies used the Traffic Management Sector-Specific Incident Case Data Report maintained by the Traffic Administration Bureau of Hunan Provincial Public Security Ministry to determine the relative contribution of illegal behaviour to motorcycle KSI crashes, conditional on real-world collisions between motorcycles and motor vehicles [86,87]. Then, ref. [88] indicated the enforcement of five laws associated with safe road behaviour (i.e., motorcycle helmet laws, national child restraint laws, national seatbelt laws, national speed laws, and national drink driving laws), GNI per capita, and the traffic fatality rate concerning each of the nations. Other studies [89,90] explored the effects of helmet laws related to any offsetting or improvement of the effects of motorcycle crashes [37,91,92]. Another study used a comprehensive crash database and analysis system via its Critical Accident Reporting Environment (CARE) [93]. The authors of [94] experimented with using a research database and provided high classification accuracy for detecting drivers’ emotions which result in smooth or aggressive driving. Furthermore, [95] presented a custom-made computer program (in a scripting language similar to BASIC) for generating DCs of MCs, SAs, and PCs from pre-processed speed profiles. Other studies provided observations about analytical factors associated with traffic signal operations [68,96,97], T-intersections [98], and traffic conflicts [99]. Furthermore, other studies focused on assess the relation between traffic injuries from motorcycle accidents and the riding behaviour of motorcycle drivers [89,100]. Other studies examined the records of fatally injured motorcyclists and collected information associated with age, sex, death cause, and time of death relative to the time of the crash. Toxicology analysis and blood alcohol concentration (BAC) results were obtained and examined, determining illegal driving behaviour as a cause of motorcycle KSI crashes and modelling crash severity by considering the risk indicators of drivers and roadways for reducing the severity of crashes and enhancing the safety performance of the traffic system [101,102,103,104,105,106,107].

### 3.2. Experimental Studies

The second major category of taxonomy involves (*n* = 41/174) studies that use an experimental approach. This section is divided into two subcategories based on data collection approaches and experimental studies. This research did not include agent-based simulation studies.

#### 3.2.1. Real-Time Field Tests

The first subcategory of experimental studies is real-time field tests. One of the studies used an instrumented Honda motorcycle with a 100-cc engine capacity for recording the real-time riding behaviour of study participants. The relation between experienced riding pleasure and riding behaviour in field tests was examined [108,109]. Other studies collected data by using a helmet with cameras measuring driver eye movements and eye fields (EMR-9, NAC) [110]. Other articles used on-board diagnostic (OBD) [111] cameras [108,112], a Xiaomi Redmi 4A smartphone application [113], accelerometers [108,112,113,114,115,116], gyroscopes [113,116], and GPS sensing [111,117].

#### 3.2.2. Experiment Using Simulation

The second subcategory of the experimental study is simulation tests. This category refers to articles that utilised a simulator for data collection to determine the factors that are related to driver behaviour [118,119,120,121], used chassis dynamometers with the standard driving cycle and onboard portable emission measurement systems in real-world driving conditions [122,123,124], the MOTORIST riding simulator [125], the mid-range simulator located at the Monash University Accident Research Centre [126], and moving-based motorcycle simulators comprising a motion platform, sound system, screen projection, and image-generation software [127,128,129,130,131]. One study attempted to estimate the impact of low BAC on the riding performance of motorcycle drivers by utilising an advanced motorcycle-riding simulator [132]. Another article made an effort to model seepage using a cellular automaton (CA)-based simulation [133]. In addition, such interactions between vehicles impact the capacity and safety of facilities [130]. Some studies indicated such behaviour in CA-based models [132]. The results showed that the software cannot replicate seepage behaviour, which was reflected in the trajectories [128,134]. In addition, driving simulators were used by other studies to understand driver behaviour [135,136,137]. In another work, the steering, braking, and throttle inputs were evaluated with the use of hardware related to a commercial steering-wheel system for gaming applications [127,133,138]. Another study used an adaptive staircase for estimating the individual driver’s gap acceptance thresholds by changing the approaching vehicle’s distance [139] and using simulator sickness, PC-Crash simulation, MADYMO [100,140,141,142], and video scenes of the motorcycle within each driving environment. Other research focused on implementing a mobile system to monitor traffic conditions (motorcycle-mounted system) [143] and initial designs regarding a simple low-cost EDR prototype using just external sensors, such as IMU, GPS, and compass information. Thus, the EDR might be easily banded and cost-effective for use on motorcycles [144,145]. In addition, the driver and motorcycle have been modelled accurately for reproducing realistically possible dynamic system behaviour. Furthermore, the bike has been modelled with 15 bodies, incorporating flexibilities in two suspension groups, whereas the virtual driver involves 15 rigid bodies [146] and 3D simulations that are perspective-correct and run at the maximum [147]. All motorcycle components were modelled accurately for recreating real inertial characteristics, experimentally acquired or provided by the manufacturer. Other types of simulation-based models are intelligent agent-based simulations, which were used to model motorcyclist driving behaviour [148].

### 3.3. Review Studies

The fourth category of taxonomy is associated with driving behaviour regarding the motorcycle domain. This category summarises the present state of motorcycle driver behaviour. Only seven of the 174 reviewed articles identified human risk factors concerning road traffic accidents amongst motorcyclists in Malaysia [149]. A clear summary of the role of moto-taxi services in offering public transport options is presented [150]. Reference [3] indicated and analysed specific drivers’ behavioural patterns impacting motorcycle mishaps through literature reviews on many aspects of riding behaviour, including the absence of alertness, visibility, and issues of speeding. The authors conducted a systematic review related to previous studies that used of driving simulators of passenger motorcycles or cars for young novice or learner drivers. Learner drivers decreased traffic infractions and/or road crashes or were shown to acquire safe skills for driving compared with the non-use of driving simulators [3].

## 4. Discussions

Following the taxonomy and full reading phase, this phase aims to discuss the salient attributes and details of the previous literature. This section is summarised into three main parts: recommendations, motivations, and challenges related to driver behaviour studies. This section is important as it shows the issues and challenges related to drivers’ behaviour that future researchers can work on for policy responses. Furthermore, the final discussion involves recommendations, representing links between previous and new research. As the earlier researchers acknowledged their limitations, their knowledge will be transforming into recommendations for other researchers pursuing a similar type of study. Thus, new researchers are expanding upon previous research to enhance the work in this domain. This section tries to answer the questions that are addressed in the introduction section of this review. By implementing analytical tables (Table 3 and Table 4), comprehensive analysis can be offered to give more insights and a greater understanding of this research.

### 4.1. Issues and Challenges

**Figure 4:**
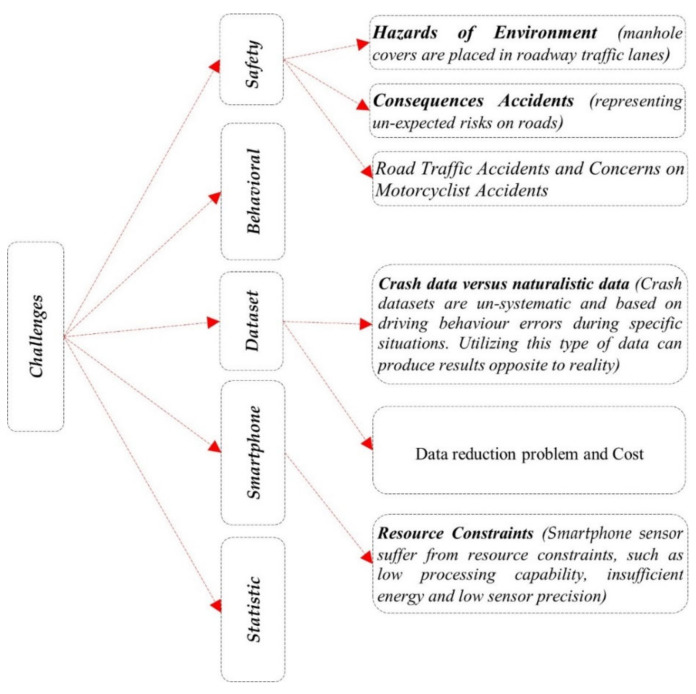
Issues and challenges overview.

The presented section describes the major challenges specified in the research of the driver’s behaviour in various domains, as indicated in the previous taxonomy (Figure 3). The challenges specified in the presented section are explained, as they come from various studies, and separating them in the presented study is too complicated. Furthermore, they were shared only based on their general significance (Figure 4), and we have grouped the challenges according to similarity and the effect caused on driving behaviour; for example, the effect of data on classification, the effect of sensors on the size of the data and data completeness and diversity, as well as hazards, such as weather conditions, that affect factors of behaviour.

#### 4.1.1. Challenges Related to Safety

The presented challenge is discussed with the key issues associated with safety concerns, as addressed in previous studies. The two most important issues are the hazards of the environment and the consequences of road accidents, and road traffic accidents and concerns of motorcyclist accidents.

##### Hazard of the Environment and the Consequences of Road Accidents

Hazards of the environment, such as manhole covers, are placed in roadway traffic lanes [81]. The consequences of road accidents might represent unexpected risks on roads and have negative effects on all society levels. Accidents harm individuals and frustrate their families, and the impact of these accidents also transcends to the general society [47]. Road traffic injuries, such as motorcycle-related injuries, are a worldwide health problem [16,151,152,153]. Reports show that social and economic road collision costs comprise approximately 5% of the gross domestic product in low- and middle-income nations [154]. The serious bodily injury and health risks for two-wheeler drivers were considerably higher compared with drivers of cars [155]. Injuries to the head and neck are the main cause of death and severe disability amongst accident victims, which also affect the prevalence of maxillofacial injuries [156].

##### Road Traffic Accidents and Concerns of Motorcyclist Accidents

Many studies focused on road crashes involving a motorcycle [56] and increased motorcycle accident fatalities [54,75,157,158,159,160], and many reported an upward trend in accident rates [15,64,110,116,161,162]. Another studies focus on traffic flow [6,114,150] is the nonexistence of efficient road facilities in advancing nations. The majority of cities in developing nations, such as India, experience serious traffics congestion because of the nonexistence of separate lanes for various vehicle classes. Thus, driving patterns related to private and public transport impact each other [95]. Other studies focused on challenges related to powered two-wheelers (PTWs), in which the truth of PTW vehicles has been their involvement in fatal and severe accidents [3,18,140,150,163]. The next challenge concerns motorcycle street racing, with motorcycle street racing becoming increasingly in Malaysia and becoming a main public concern [10]. Another challenge is weaving, creating turbulence, impacting progress, and resulting in low capacity and creating bottlenecks in road systems [96]. The leading cause of death in road traffic accidents [164] is the increased injuries sustained in road crashes [8,12,26,42,66,74,75,118,152,154,164,165,166,167,168,169,170,171,172]. Previous studies indicated that approximately one-quarter of mortalities from traffic accidents involved motorcycle drivers [33,47,51,159,173,174].

#### 4.1.2. Challenges Related to Factors of Behaviour

Considerable research studies have linked aggressive driving behaviour with accidents [39]. Aggressive driving has been associated with elevated risks of car crashes [63,175]. Anger is also considered a significant human factor in causing road accidents. A few comparative studies between low- and high-danger drivers have been conducted. The results indicate that high-anger drivers commit more traffic infractions compared with low-anger drivers [176]. Night driving is considered a challenge for driving simulations [128]. Several riding behaviours which lead to injuries and crashes have been indicated. However, correcting these is highly challenging [12,177].

#### 4.1.3. Dataset Issues

One of the distinctive challenges faced by traffic-movement researchers was the lack of the required adequate experiential data for studying motorcycle driver behaviour, leading to only the designing of experimental models. Therefore, such a research area’s sluggish development is still an issue [178]. Expired data and incomplete, short time periods provide insufficient style representations [118,119]. The incomplete datasets affect the adjustment process’ accuracy. Creating a precise model to understand the behaviour of drivers is complicated. Features shaping the behaviour of drivers have not been recognised. Data on features of drivers’ behaviour have not been adequately diverse in representing the styling of driving, and real-time monitoring involves various errors [26,60,68,111,114,179,180,181]. Using certain fields concerning driving behaviour investigations might result in biased datasets [26,60,68,111,114,179,180,181]. Furthermore, the experimental datasets from simulators have been unrealistic with few motorcycles or limited sample sizes [160].

##### Crash Data versus Naturalistic Data

Driver behaviour classification models utilise crash datasets focusing on quantitative numerical methods. In addition, safety level representations might be recognised after some critical situations. The crash data have some issues, such as consistency, size, diversity, and length of the process of data collection [182]. The crash datasets were un-systematic and based on driving behaviour errors throughout certain situations. The use of such data might provide results that contradict reality [183].

##### Data Reduction Problem and Cost

A problem is encountered in reducing the data size. Manual approaches require costly efforts. However, computerised methods achieve excellent results within specific conditions. Even expensive, resilient systems produce errors and require the supervision of humans for authenticating the results [26,60,68,111,114,179,180,181]. In addition, data collection and size reduction were the two main procedures vital for describing driver behaviour. However, the approaches to performing such processes were costly, thereby limiting the time, location, and distance of the experimental study [85].

#### 4.1.4. Smartphone Sensor Issues

Smartphones have been utilised for collecting data on motorcycle driver behaviour. Such devices suffer from resource constraints, including low precision of sensors, inadequate energy, and low capability for processing. Moreover, the raw data acquired utilising smartphone sensors suffer from sparsity [157]. Using smartphones and other systems, including OBD, causes an increase in effort and the need to fit or connect more devices. Therefore, the system will be less secure, unscalable, and impractical [111]. Studies based on smartphone usage handle single-perspective driver behaviour properties; therefore, they suffer from bias evaluation and integrity issues [144]. Furthermore, the data acquired from smartphones show issues associated with integrity, reliability, and validity caused by using embedded low costs and undependable sensors. Numerical investigations and data mining are achieved offline, assuming vast amounts of gathered data [19].

#### 4.1.5. Static Sensor Issues

Fixed-point sensors (such as loop detectors and surveillance cameras) have been employed in several academic studies, although this method of data collection has several flaws. Sensors, for example, are fixed in place and cannot be relocated; changing their placement and updating them regularly requires a significant amount of effort. As a result, data is collected from fewer and less diverse sites. Another issue that affects the quality of data collected by loop detectors is the inaccuracy caused by the incorrect type of velocity identification, as well as the moving physical objects that cross these sensors. Surveillance cameras are another sort of fixed-point sensor; they collect data from a restricted number of sites and are expensive to install and maintain. These cameras also produce various mistakes as a result of shaking, congested traffic, regularly blocked observations, lighting variations [2].

### 4.2. Motivation

**Figure 5:**
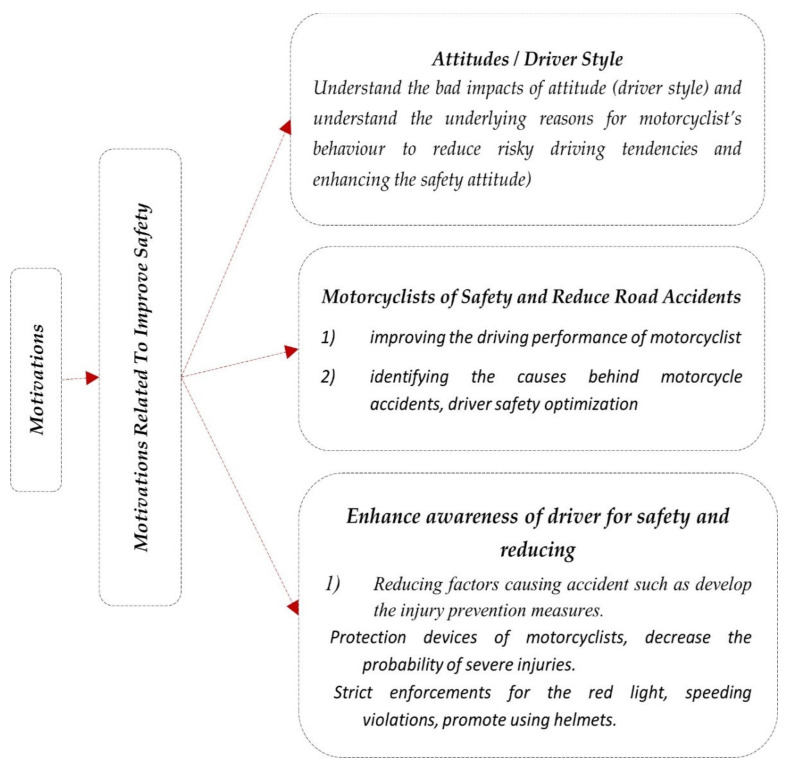
Motivation overview.

This section describes the literature that focused on adapting motorcycle driver behaviour over various domains. The motivations have been categorised based on similarities and general purposes (Figure 5).

#### Motivations Related to Improving Safety

Analysing and knowing the behaviour of motorcycle drivers is an important aspect of improving safety. Researchers are encouraged to pursue such types of research for various reasons. The reasons include understanding the attitude of motorcyclists towards their safety in addition to the road’s safety, understanding why young motorcyclists are involved in several collisions [56], and reducing road accidents [51,76,153,157,170,180,184]. Another reason is to improve the driving performance of motorcyclists, identify the causes behind motorcycle accidents [50,157], promote road safety [25,38,54,76,94,121,152,154,185,186], reduce motorcycle fatalities [108,159], avoid accidents [51,110], and improve road safety education amongst all motorcyclists [53,99]. Others looked into safety from different perspectives, such as improved traffic system safety [18,187,188] and motorcycle safety [76,78,92,97,119,123,127,162,165,173,179,180,189,190,191,192]. Moreover, effective hazard detection is critical for hazard elimination and accident consequence reduction [81,118], the identification of possible hazards in their early stages before causing accidents [155], and the reduction in the probability of severe injuries [84,98,139,151]. Other researchers explored the severe impacts of attitude (driver style) to understand the underlying reasons for motorcyclists’ behaviour [42,75,193]. To reduce dangerous driving tendencies and enhance the safety attitudes of young drivers [116,164], understanding risky riding behaviour amongst motorcycle drivers is imperative [194]. A few researchers focused on enhancing drivers’ awareness of safety and reducing factors that cause accidents [161]. The assessment of driver information systems [121] enhance driver awareness [143], reduce teenager crashes in addition to the severity of their injuries [172], improve self-motion perception [131], provide suitable street lighting and road delineation, strict enforcements for red light and speeding violations, promote the use of helmets and enhance motorcyclists’ vision [179,189,195].

### 4.3. Recommendations

**Figure 6:**
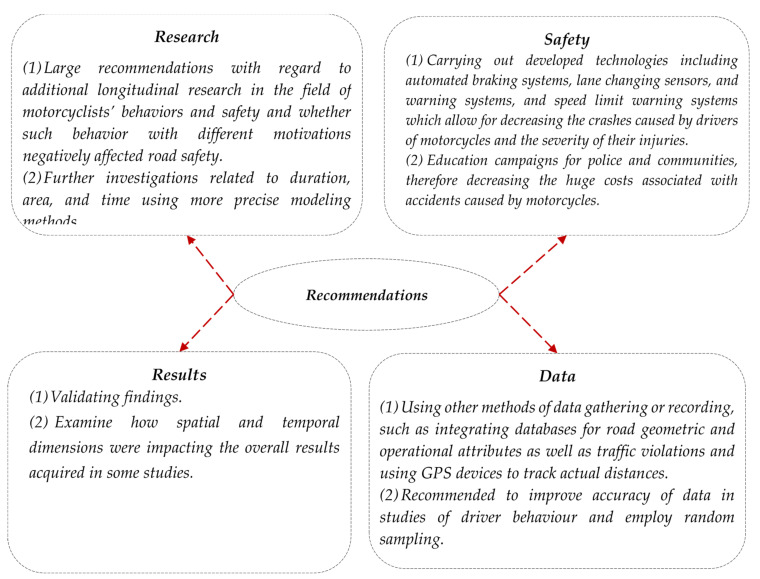
Recommendations overview.

This section discusses the literature with recommendations related to previous research for future directions (Figure 6).

#### 4.3.1. Research Recommendations

This section highlights clear recommendations for future works. The reviewed studies provided a number of recommendations on areas such as complimentary research work [39,54,67,78,98,114,115,121,185], drivers’ behaviour [139], duration [16,62], area [3], time [84,196], and the use of more precise modelling methods [197]. Many researchers pointed research direction towards longitudinal research in the field of motorcyclists’ behaviour and safety and whether such behaviour with different motivations negatively affected road safety [10,63,139]. This section provides some sample studies that offered specific recommendations. The next group of researchers suggested more research on risk reduction counselling efficiency, which should be aligned with drivers’ post-crash impressions [6,12,116]. Another group of researchers recommended further investigation into the relationships between collisions and the various conflict indicators [99,173].

#### 4.3.2. Recommendations Related to Safety

Researchers recognise the significance of safety in most research and offered their views and ideas for future works. Future research attention is drawn towards road safety in motorcycle driver behaviour studies. Examples include the following: finding the effective ways to improve road safety [165], installing motorcycle-friendly roadside barriers to protect from hitting light poles and trees [192], strengthening the traffic laws and regulations along with the traffic safety education by creating awareness of road safety through different public media [25,33,89,190], developing simulation models [3,98,163], protecting the motorcycle driver [25,33,161,164,198] reducing accidents [89,123,187,189,199], avoiding fatal injuries [19,53,100,151] and increasing road safety by predicting the driving performances [51,153,200]. However, some scholars suggested developing limited warning systems to reduce accidents [172]. The other researchers also suggested supplying helmets of suitable quality, along with major education campaigns for police and community, thereby decreasing the large costs associated with accidents caused by motorcycles [115,143].

#### 4.3.3. Recommendations Related to Related Data

Data are an essential element of research. Some studies recommended more data collection by using other statistical methods of data collection [118,119] and integrating databases of road geometric and operational attributes and traffic violations [160]. Data can be obtained by either web- or application-based surveys [66], by using GPS devices to track actual distances [15], and by providing more drivers’ information [168]. Other studies suggested more research on longitudinal data and the use of different data sources, such as experimental data in real-time by using smartphone applications or sensors [26,60,68,111,114,179,180,181]. Many scholars encouraged improving data accuracy in studies of driver behaviour [65,160] and employing random sampling [66].

#### 4.3.4. Recommendations Related to Findings

This section presents recommendations for the verification of results and the evaluation aspect of some methods used in previous research. Researchers should focus on results validation [16,92]. Furthermore, examining how the spatial and temporal dimensions impact the overall results acquired in some studies might be significant [160], and the findings should be treated with absolute care [49,84]. Other recommendations included comparable results [131,171].

## 5. Methodological Aspects of Previous Research

**Figure 7:**
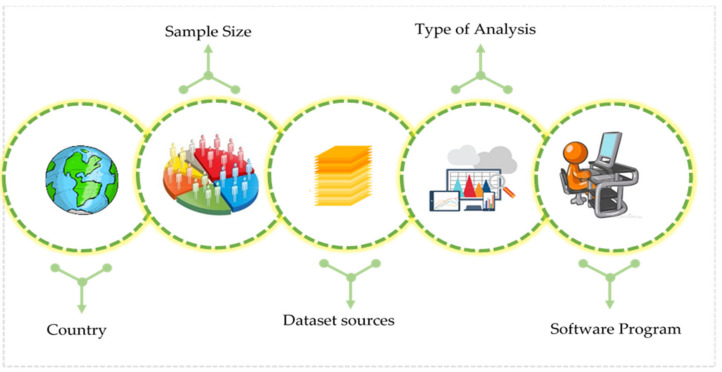
Methodological aspect overview.

The section explains the methodological approaches used in previous research. Many attributes were covered in this section, and all attributes will be considered, as shown in (Figure 7).

The importance of reviewing previous research modelling techniques is evident from the previous study suggestions. For instance, future research on motorcycle driver behaviour should be carried out by identifying well-designated areas with the correct sample size and method of analysis. Moreover, future researchers should compare findings across countries. The analysis software, the data sources utilised, and the provided approaches should be well-designed.

### 5.1. Country

**Figure 8:**
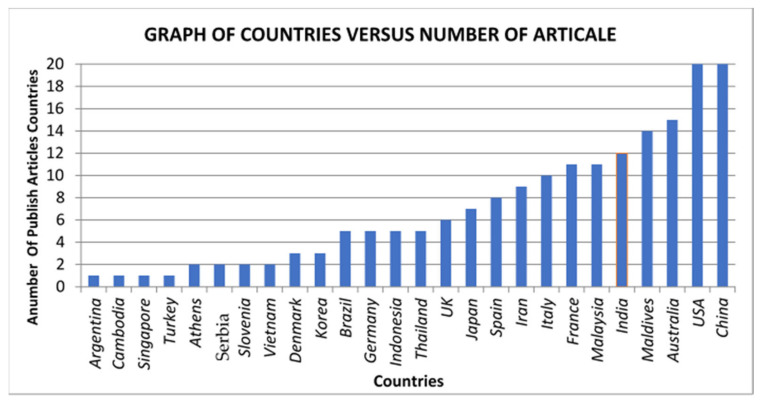
Numbers of included articles based on countries of origin.

Globally, considerable attention has been given to drivers of motorcycles. Thus, such research is of high interest for the agencies and research institutes that utilise such studies for research and social, medical, and other purposes. The present work indicates that the majority of studies on the behaviour of motorcycle drivers are from 32 countries, with the United States of America, Australia, and Malaysia having the most published works (Figure 8).

Figure 8 depicts the nation of author affiliation (the author’s country has been specified in studies with multiple authors). Most of the motorcycle studies were in Australia, China, and the USA. Other categories originated from Greece, Denmark, Maldives, Argentina, Serbia, Cambodia, Nigeria, Norway, Singapore, Turkey, Venezuela and Colombia, with one article from each nation. In other countries, such as China, Brazil, Germany, Malaysia and Vietnam, the number of studies ranges from 2 to 15. Table 5 shows Countries, with references.

Only 26 out of world nations carried out studies on motorcycle driver behaviour. Thus, various gaps in academic efforts exist in the remaining countries [51,110], specifically Southeast Asia. Another area of academics that has been rarely explored is models comparing the driving behaviour of motorcyclists [198,212]

### 5.2. Sample Size

**Figure 9:**
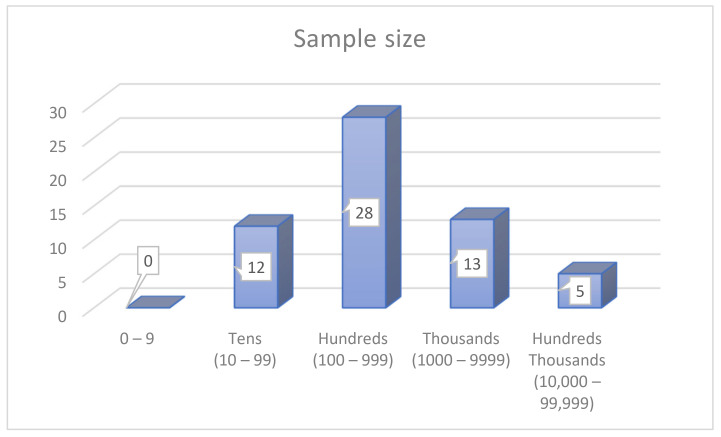
Sample sizes used in studies using social science techniques.

With the sample size, we represent a subject drawn from the whole population, which is considered a subset of the entire population of a specific research endeavour. This current section highlights the methodological aspects and sample size of previous studies of motorcycle driver behaviour. In addition, the number of participants varied between studies. This section presents all previous sample sizes applied by respective researchers, which will be categorised into three categories. The first category, sample size based on social science techniques (Figure 9), focuses on using four groups of different sample sizes. The size of the first group is in the tens and ranges between 10 and 99 participants [12,15,25,75,88,94,121,124,176,191,197,204]. The second group is in the hundreds and ranges between 100 and 999 participants [8,10,27,33,38,39,40,42,53,54,56,58,60,66,100,119,152,153,156,173,184,186,198,207,210,219,236,241]. The third group is in the thousands, and ranges between 1000 and 9999 participants [16,17,18,65,76,80,151,160,161,164,180,181,242]. The fourth group is in the tens of thousands and ranges between 10,000 and 99,999 participants [84,172,174,179,194].

**Figure 10:**
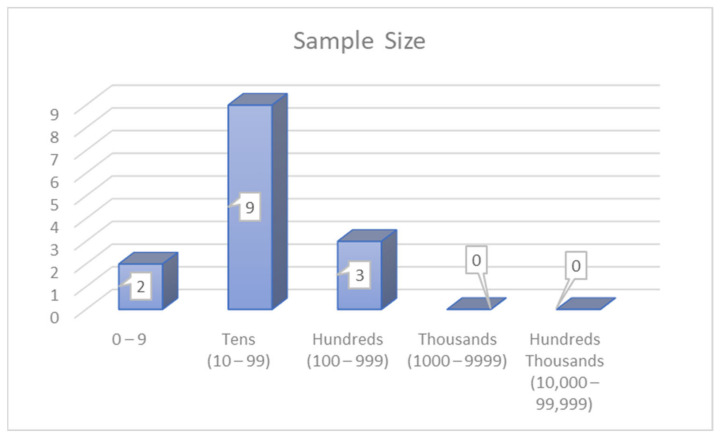
Sample sizes used in studies using simulator techniques.

The second category is based on sample size based on simulator techniques (Figure 10). This category is divided into two subgroups of sample size. The first group is the tens, which used between 10 and 99 participants [6,123,127,128,131,141,154,194,200]. The second group is the hundreds, which utilises samples between 100 and 999 participants [51,133,237].

**Figure 11:**
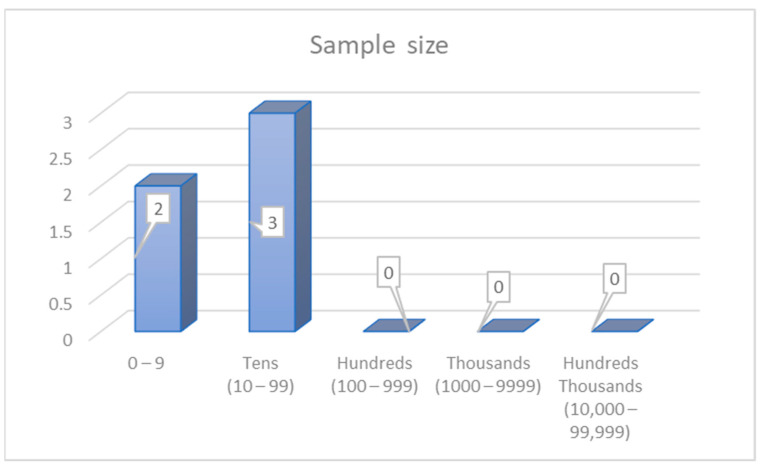
Sample sizes used in studies using real-time field tests.

*The third category is sample size based onreal-time field* tests (Figure 11). Two sample size groups exist. The first group used between 0 and 9 participants [62,108,110]. The second group consists of a study that used between 10 and 99 participants [114,130]. All samples have been used across various studies. A few studies have been utilised more often compared with the other group of participants.

A large sample size offers reliable and accurate data and gives more insight into the driver’s behaviour. However, this sample size is associated with selecting precise tools for data collection and the time needed for conducting the experimental study and analysing and pre-processing the data.

### 5.3. Dataset Sources

The data are an essential element of high-quality research for all studies on motorcycle driver behaviour and have been a major significant aspect as the liaison between findings and analysis. The authors used several datasets to determine motorcycle driver behaviours and their relation to road accidents. The data sources have also been indicated in various studies from different sources using the previous literature in driver behaviour studies Table 5. The first source is a survey through interviews and questionnaires, as revealed in the literature. Others obtained data from medical and care centre institutions, including hospitals, medical systems, records, centres, and clinics. Some researchers obtained data from the *Department of Transportation (NMDOT) Traffic Safety Division and police reports*. Other researchers depended on data that were provided through experimental means and observation. Some studies depended on a number of data sources for motorcycle driver behaviour in real-time, such as video cameras, trials, and recruitment. The last group of researchers outsourced from other places that involve online resources, including scientific databases, websites, safety and information systems and panel data. Knowing the data source enables the researcher to understand how data across different academic literature were gathered. The researcher can understand his/her data source options and determine whether the available data at their disposal is academically suited or not. Table 6 shows the references of previous data sources.

### 5.4. Type of Analysis

This section presents an overview of the types of analysis utilised in driver behaviour studies, as summarised in Table 7. Many articles used descriptive statistics, sensitivity analyses, empirical analyses, qualitative analyses, confirmatory factor analyses, data distribution analyses, path analyses, in-depth analyses, meta-analyses, and exploratory factor analyses. Different types of analysis were used, including regression analysis, multivariate linear regression analysis, binary logistic regression analysis, multiple regression analysis, and headway analysis. A detailed motorcycle accident data analysis was carried out by statistical analysis, and an odds ratios analysis has been applied for exploring the impact of single variables on cycling and driving aggression while simultaneously controlling the impact of other variables, such as cycling and driving frequency. The following techniques are used less frequently: automated video-based analysis techniques, linear regression analyses, and reference analyses. The latter is related to the other analysis types that have few occurrences in the literature and include Bayesian-related analysis, vibration analysis, macro and microanalyses, cross-sectional analysis, and chi-square analyses. Table 5 shows the types of analysis with references.

It can be pointed out from Table 6, that the authors used different types of analysis to describe the driving behaviour of motorcyclists. The adopted analysis techniques varied among the previous literature work. Each author adopts the type of analysis that he/she believes will provide valuable insights. The number of analysis methods adopted in each article was limited to one type only. There is no implementation of multiple types of analysis in one work. Some articles applied descriptive analysis only, with no inclusion of another type of analysis such as linear regression. Furthermore, the number of factors and parameters included in each analysis also varied among previous research work with no agreement on the most effective factors or variables that can describe driving styles in detail.

### 5.5. Types of Software Programs

This section provides an overview of previous studies’ use of software. The SPSS is the majorly utilised software in the motorcycle driver behaviour studies considered for data analysis [10,58,79,236]. *MATLAB* is the next most applied software [62,80,114,139,146] followed by test software or testing procedures [94,121,131,148,156,186], *Microsoft Excel* [181], and *Python* [190]. Previous studies focused primarily on SPSS and *MATLAB* compared with Python because *Python* needs a high level of programming skill for data analysis.

## 6. Substantial Analysis

This section describes the substantial analysis performed on the related work. This analysis is categorised into four parts. It comprises analyses of real-time-based articles, dataset labelling procedures, data acquisition systems (DAS), and the inclusion of environmental factors.

### 6.1. Analysis of Real-Time-Based Articles

This section presents analyses of the motorcycle experiments conducted in real-time conditions using different types of sensors. It can be seen from Table 3, that different studies used a different number of features in their studies. Speed features being the most used feature, followed by acceleration, but less consideration was given to other features such as right turns, left turns, etc., and sleep and fatigue factors. Distraction features were also not considered in motorcycle driver behaviour modelling. The gender factor was not considered during the real-time experiments. For machine learning-based models, the selection of features was mostly based on a correlation map with no use of the special algorithm for automatically selecting the effective features. The real-time studies presented in this table are mostly small-scale studies based on the number of drivers participating in each experiment and the length of the experiment, except for one study which was conducted in an urban and highway environment (78 km road length), which is considered a large-scale study. It is recommended that authors implement different types of studies (especially large-scale) to measure fatigue and stress levels while driving. A framework for motorcyclist behaviour in large- and small-scale driving conditions is still lacking.

The age factor was not considered in most of the studies. The pre-processing step of missing data was not reported fully in the experimental studies. The treatment procedure for missing data is not known, as the examination of missing data is yet to be performed, especially examining its effect on data labelling and classification accuracy. From Table 6, it can be pointed out that most of the machine learning-based articles used multiclass classifications, except for one article that used binary classification. However, there is no mention of the problem of unbalanced data, the problem of insufficient data, or data bias as well-known problems for multi-class classification techniques. Multi-class classification is a complex well-known procedure with many issues. Class separability, class overlaps, and imbalances between and within classes are the most mentioned problems for multi-class classifications [243]. Patterns that are nonlinear and unseen in the dataset can add more complexity to multi-class categorisation [244].

Also, there is no agreement on the number of machine learning algorithms that should be applied for behaviour modelling. It can be seen from Table 8 that most of the authors collected their data using specially designed equipment, i.e., data acquisition systems (DAS), or using a smartphone, indicating that there is no free, accessible data that is available for use.

Accordingly, this increases the cost of the experiment and decreases the level of willingness to conduct driver behaviour modelling. Statistical analysis techniques are widely used in real-time studies. This indicates that this research area is yet to recognise the role of data-driven methods for driver-style recognition or prediction. Using social science-based methods or quantitative methods does not reflect the actual data that represents motorcyclist behaviour. This type of method is biased and uses a simple perspective of parameter interpretation, as it assumes that there is a rule that defines the relationship between the parameters that represent the driving style. Simulator-based methods are conducted in a controlled environment that do not reflect actual driving conditions. It can be pointed out from this section’s analysis that statistical-based methods of analysis are more inclusive of human factors, rather than artificial intelligence-based models. Real-time experiments based on an instrumented motorcycle can provide the most accurate data; however, this is costly and needs special arrangements for the safety of the motorcyclist.

### 6.2. Dataset Labelling Procedure

This section discusses the labelling procedure that has been followed by previous research articles. Data labelling is the process of identifying the collected data with a descriptive name to describe its category. Labelling is a data pre-processing technique for driver motorcyclist behaviour recognition. Two main methods have been proposed in the literature for labelling datasets. Namely, manual (pre-post) testing. In this technique, a normal experiment is conducted to collect normal behavioural data and then an abnormal experiment is conducted to collect abnormal behavioural data [110]. The authors of [113] used manual techniques to mark the behaviour of motorcyclists. Moreover, the authors of [116] used manual techniques by using thresholds and rules for data labelling. The second method is the automated method, which was implemented by the authors of [114], who used the proprietary software BinAscii for data labelling. However, the manual labelling technique suffers from bias and is not accurate. For instance, it is tough to label stress, since the consequences of events are seen differently based on the user’s profile and current state. Furthermore, when manually identifying problematic zones after driving, drivers tend to forget about stressful events. Physiological signals, on the other hand, are very susceptible to noise and serve merely as approximations for assessing human mental states [116]. For automated labelling, the data is automatically labelled without human interference; however, the labelling process is performed using rules and equations that have been designed for other case scenarios in a different area with a different purpose. Furthermore, the expert labelling may be biased or inaccurate. The literature encourages the classification of motorcyclist driving behaviour into multi-type driving styles recognition rather than binary classification. However, a consensus is raised on the issue of labelling and how to attain the best technique (expert labelling or questionnaire, automated, unsupervised machine learning-based, etc.). Moreover, there is no agreement on the best method of labelling. Unsupervised learning is yet to be considered for the labelling process. It is recommended to investigate the efficiency of unsupervised learning algorithms for the data labelling process, i.e., K nearest neighbours is a widely advised machine learning technique for this task, but careful tuning is advised, especially when selecting the K number.

### 6.3. Data Acquisition Systems (DAS)

This section presents the sensors used in real-time motorcycle driver behaviour analyses. Table 7 points out the types of sensors used to examine motorcycle driver behaviour in real-time situations. It can be seen that a different number of criteria are used to evaluate the DAS used for data collection in real-time experiments. Installed DAS, special modifications, complexity, cost efficiency, and the reliability of the DAS are the used criteria in DAS assessment. It can be illustrated that there is no agreement on the number or type of sensors used in DAS designs, as each study customises the data collection system to fits its experiment’s need. No generalised framework has been presented on how to select the best sensor/DAS to collect the data. The data collected from motorcycles using CAN-BUS is observed in three articles, indicating limited usage. Hence, motorcycles’ actual features are not provided accurately in most studies. Moreover, there is no indication of using range sensors such as LiDAR or radar. These sensors are necessary for measuring the distance to cars and other vehicles. Furthermore, there are only two articles that used smartphones for data collection. Hence, a framework that compares the performance of DAS is needed, especially in terms of the size of the missing data. A smartphone-based data collection system is a cost-efficient way to collect data. However, the smartphone is well-known for its resource constraints. Furthermore, it has poor performance regarding data sparsity. Its sensors are not reliable and generate missing data most of the time. The DAS in Table 7, have been evaluated based on their components and the method of their working principles. Since there is no standard design for DAS, many authors designed their own DAS with specific customisations to satisfy the needs of their experiment. Accordingly, some DAS require special modifications for motorcycles to be fit into. Hence, increasing the complexity decreases the cost-efficiency. These subjective evaluation values give more insights regarding special modification, design complexity, and cost-efficiency. These three criteria are necessary to assess a system’s structure and to give insights as to what extent it can be implemented for a large number of motorcycles (platoon experiment). Moreover, it sheds light on design planning for future research, as researchers can have a basic idea about the DAS framework in terms of electronic sensors, systems, and data specification (diversity, size, completeness).

### 6.4. Inclusion of Environmental Factors

It can be pointed out from Table 4 that a different number of studies used different road types (highway and urban). However, no study considered suburban or mountain environments. The real-time experiments were conducted at different times of the day, with no reports on experiments during the night. One experiment was only conducted during hazardous weather. The literature lacks a complete framework for experimenting during different times and in different weather conditions. Furthermore, there is no agreement on the length of the road, as each experiment was conducted using a different length. Most small-scale studies were conducted using road lengths between 5 and 20 km. Moreover, naturalistic studies (over months) are not reported in the literature. The literature is also missing an experiment that measures driver behaviour during car-following or motorcycle-following in real-time conditions. These experiments are necessary to measure important aspects of motorcycle driver behaviour.

## 7. Comparison between Our Work and Previous Academic Work

This study presents a comprehensive review conducted on various but related research topics concerning motorcycle driver behaviour. As far as we know, no survey articles or reviews cover the model regarding motorcycle driver behaviour from the perspective of data sources or provide a thorough analysis related to data collection with the use of instrumented motorcycles driving in real-time fields. The existing literature does not offer enough knowledge to evaluate the presently suggested/used classification model’s performance. In addition, the number of researchers exploring the classification model’s functionality, cost efficiency, and complexity was lacking. Choosing optimum cost-effective/practical DAS was complicated because of the lack of clear criteria concerning performance evaluation. Therefore, this work aims to analyse the literature associated with motorcycle rider models, types of data, and systematically searched related articles. Table 9 shows the differences between the presented study and previous ones. The action point-based motorcycle driving behaviour, data-driven, deterministic models were covered based on availability in collected articles. Road safety and traffic management were surveyed, specifying the requirement for enhancing the developments in such areas of research. The use of the artificial intelligence model for motorcycle recognition is illustrated and examined. The labelling process of collected data is also investigated along with classification algorithms. Classification and recognition problems were addressed based on the availability of these topics in collected articles. A recommendation section is provided for future studies.

## 8. Towards Intelligent Transportation Systems

A potential research direction might be provided based on our understanding of the literature associated with motorcycle driver behaviour. Many points were taken into account for future developments in such a research field.

### 8.1. Optimal Sensor Selection to Collect Data

The sensors’ types might be chosen according to current sensors’ capability to record and sense entities in real-time traffic conditions [237]. The used sensors must assess the actions of drivers (lane changing, reversing, and angle tuning), speed differences, conditions of traffic (low, medium, or high), and the status of the driver (distracted, heavy-eyed, focused, hostile, or sleepy) [5]. Such metrics might offer a whole picture regarding the style of driving and identifying the critical driving conditions [7]. Moreover, an approach to selecting a combination of adequate sensors was required to detect and record all features/factors [159]. One of the questions raised regarding sensors is that image detectors are less effective at carrying out in-depth measurements and distinguishing objects than LiDAR and/or radar. Radar sensors present the drawbacks regarding low lateral-resolution data. Even though LiDAR sensors can overcome radar and image sensors’ limitations, they show issues associated with their capability for distinguishing objects, latency identification, and clustering errors [246]. Accordingly, on-board diagnostics (OBD) require data collected just throughout off-peak hours because of safety concerns. The rider should be carrying a bulky load that contains devices and a power source that rests on the pillion seat of the vehicle [111]. Additional vehicles require a line of sight and assume 100% reflectivity, excellent weather conditions, and an even road surface. The author of [27] used Automatic Idling Stop and Go (AISG) technology because of the differences between automobiles and scooters concerning driving behaviour. The AISG topology, as well as the control strategy utilised in automobiles, cannot be used for motorcycles directly with on-board diagnostics (OBD) [111], accelerometers [114,115,116], or gyroscopes. The researchers pointed out that the accelerometer and gyroscope are very noisy and thus require filtering [14]. Hence, more research is needed to measure the effectiveness of sensors in detecting motorcyclist behaviour. A general framework to describe the procedure of selection and benchmarking is needed to help identify the equipment that best captures driving behaviour, whether it is a sensor or a DAS in different conditions and at different times. The framework should consider the trade-off between cost-efficiency, complexity, and scalability criteria.

### 8.2. New Methods and Datasets

Future research on driving behaviour could consider technological advancements for measuring driving time and distance, crash risks, and driving skills. Furthermore, smartphone sensor apps might show significant possibilities for assessing motorcycle driver behaviour. Moreover, future research directions are to investigate the effect of missing data on the classification and recognition of motorcycle driver behaviour. A complete framework that conducts real-time experiments on different road types, at different times of the day, in different weather conditions, and during different traffic statuses is recommended to give more insights into motorcycle driver behaviour. The use of deep learning techniques is also recommended, though this may require a lot of datasets; augmentation pre-processes and other data maximisation techniques can be examined to test its capabilities to enhance the recognition models. Furthermore, more studies are required to examine the effects of unbalanced datasets on the model accuracy and labelling procedure. Missing data treatment in the pre-processing step is a recommended future direction. The impact of missing data on the labelling procedure and classification accuracy is advised to be investigated. A framework for selecting the best machine learning that can impute the missing data is advisable. Another aspect for future directions is sample size identification. More elaboration on the sample sizes required to be included in real-time experimental studies is needed. Identifying the number of observations is a great advantage for small-scale, cost-efficient, real-time-based studies

### 8.3. Availability of Data Collection System

In this field of research, real-time data is essential for progress. Additional data can help with modelling and comprehension. The most significant constraint on dataset availability is the lack of cost-effective and scalable technology for collecting data reliably and quickly [6]. The construction and development of DAS are expensive, especially for long-term experiments or short-term research with a large population sample and multiple motorcyclists employed for data collection. As a result, delivering a low-cost, dependable, and simple-to-implement system is a huge step forward in the research. To increase road traffic safety in ITS, motorcyclist behaviour prediction approaches using online sensors in real-time situations are required. Moreover, sensor manufacturing companies and system design developers should consider designing more flexible systems able to easily integrate multiple sensors without the need to implement soft interfacing procedures between the sensor and the main board of the data collection system. This step can decrease the level of complexity in DAS and increase the researcher’s willingness to implement more real-time experiments using instrumented motorcycles. Moreover, the designers and manufacturers should consider a level of reliability for the data collection system, as a cost-efficient system does not necessarily mean integrating low-cost and less reliable sensors. For example, power protection circuits and reverse polarity protection circuits can be included in the design of a low-cost microcontroller board as they provide more protection and system reliability.

### 8.4. Selecting the Best Algorithm for Driving Style Recognition

This section addresses concerns and problems with machine learning-based models in terms of assessment, benchmarking, and selecting the best machine learning-based model. Benchmarking is the process of comparing a freshly constructed model to existing models utilizing similar conditions and characteristics. Examining the performance of motorcycle models to reflect real-world driving behaviour is one of the strategies used in assessment and benchmarking. Several factors must be considered while developing new machine learning-based models, including a low mistake rate, high reliability, minimal complexity, and high accuracy. However, meeting one of these requirements but not the others will have an impact on actual performance. To highlight performance in real-world applications, these factors must be thoroughly studied. Future studies should take into account potential conflicts or trade-offs between these criteria or measures, and a clear, reliable mechanism should be developed to resolve this potential conflict. During the evaluation and benchmarking processes to test and compare the performance of the developed machine learning-based motorcycle driver behaviour models, the following evaluation criteria for machine learning-based models must be considered: accuracy, precision, true-positive rate, false-positive rate, true-negative rate, F-measure, training time, area under the curve, and error rate. The focus of the research should be whether the created machine learning-based motorcycle driver behaviour models take into account all of the evaluation and benchmarking criteria during development. Researchers should also look into how benchmarking and assessment are handled in developed models [2].

### 8.5. DAS Evaluation Procedure

This section provides some highlights on specific topics to address the current issues and possible solutions in future research. The selection of the best DAS that can be fitted in a specific design is still a gap that needs to be considered for future research. The cost and complexity of DAS are important to consider to make significant advancements in this research direction. However, considering one design criterion and neglecting other criteria is not the correct path for DAS selection. Table 7 presents subjective evaluation values for criteria that describe the DAS as a system from the perspective of researchers. It can be pointed out that there is a conflict and trade-off among the evaluation criteria. When the cost-efficient criterion is high, the complexity level and DAS reliability are low. This trade-off affects, in one way or another, the scale of the research design, whether it is small-scale (short time with limited sample size (<30)) or large-scale (NDS). For a more reliable and comprehensive DAS assessment, all assessment criteria should be considered in the evaluation process of the DAS. Using different weighting procedures, the criteria can be given an important value to incorporate a level of accuracy in the assessment procedure. Hence, comparing (benchmarking) multiple DAS with each other can have better insights into the strengths and weaknesses of each proposed DAS. to select the best available DAS, the selection procedure can be considered a complex problem that includes multiple attributes with different DAS available in the literature [2].

## 9. Limitations

Even though the presented study’s database sources covered wide groups and were reliable, identification is still complicated. In addition, a drawback of the review’s timeliness resulted from an increase in this area’s progress. Research in specific periods in such an essential field does not essentially indicate actual influence or usage. Yet, the information shows the research community’s response to the field.

## 10. Conclusions

This systematic review article surveyed the past literature related to motorcycle driver behaviour. A taxonomy for the literature was deployed to group the articles according to similarities and possible patterns to make them easy to analyse and to extract valuable insights. Key points and important findings were presented from the investigation of the motivations that keep researchers’ continued interest in this research area. Furthermore, other aspects were highlighted and illustrated from challenges, problems, and issues. Moreover, recommendation topics were described for different entities to further enhance the development in this research area. Methodological and substantial analyses are two types of analyses presented to give important insights. The development articles surveyed in this review were examined and further analysis was produced. The analysis of previous literature regarding development articles was either based on questionnaires or real-time and on-road driving using a sensory data acquisition system (DAS). Questionnaire-based models suffer from biased and questionable results. On the other hand, real-time-based experiments are more accurate but require special electronic designs and a considerable cost that cannot be neglected and which affects progress in this research area. Model representation using real-time, on-road datasets with intelligent (machine learning) algorithms is still limited in the literature. This method requires a large dataset to efficiently replicate the driving style and special electronic design systems. Furthermore, artificial intelligence based algorithms suffer from overfitting and require special tools/hardware to process the datasets. Motorcyclist driving style accuracy is mainly affected by dataset availability, reliability, completeness, and the type of implemented DAS. The selection of a DAS that fits the required design is a multi-attributed problem that needs to be considered. No generalised framework can represent the driving style of a motorcyclist at all times and in different conditions. Future research directions towards ITS were presented, with topics that hold valuable information. This systematic literature review concludes that there is no available model that has the quality to represent driving styles for different drivers in different regions or at different ages. This review gives important aspects for researchers, summarising a literature analysis and identifying research gaps.

## Figures and Tables

**Table 1:** Workflow procedure.

Attribute	Digital Library			
	Science Direct	IEEE	Scopus	Web of Science
Years	2011–2021	2011–2021	2011–2021	2011–2021
Language	Only English	Only English	Only English	Only English
Run-on	Full Text	Full Text	Full Text	Full Text
Subject Areas	All available	All available	All available	All available
Date of running/updating search string	2021	2021	2021	2021

**Table 2:** Questionnaire techniques.

Questionnaires	References	Total
Buss And Perry Aggression Questionnaire	[10,11,12,13,14]	5
Questionnaire (Demographic Information)	[15,16,17,18,19,20,21,22]	8
Road Safety Perception Questionnaire (RSPQ)	[23]	1
Social Norms Questionnaire	[8]	1
Motorcycle Taxi Drivers and Non-Occupational Motorcyclists	[15]	1
Driver Distractive Compensatory Beliefs (DDCB)	[24]	1
Self-Administered Questionnaire	[25,26,27,28,29,30,31,32]	8
Standard Questionnaire	[33,34,35]	1
The Questionnaire Battery	[36]	1
Driving Behaviour Questionnaire (Dbq)	[8,37]	14
Drivers Angry Thoughts Questionnaire (Datq)	[38,39]	2
Theory Of Planned Behavior (TPB) Questionnaire	[40,41]	2
Motorcycle Rider Behaviour Questionnaire (Mrbq)	[42,43,44,45,46,47,48,49,50,51]	11
Manchester Driving Behavior Questionnaire (Mdbq)	[52]	1
Type-A Personality Questionnaire	[17]	1
Questionnaires Motorcycle Taxi Drivers	[53]	1
The Motorcycle Safety Foundation Rider Survey Questionnaire	[54]	1
NEO-FFI-3 Questionnaire	[54]	1
Dula Dangerous Driving Index Questionnaire	[54]	1
Frequency Of Risky Behavior	[54]	1
Barkley Adult ADHD Rating Scale-IV Questionnaire	[54]	1
Web-Based Questionnaire	[55]	1
Anonymous Questionnaire	[56]	1
The Driving Cognitions Questionnaire	[57]	1
Self-Reported Questionnaire	[12,20,58,59,60,61,62,63,64,65,66,67]	12
Motorcyclists Profiling Questionnaire (MOPROQ)	[68]	1
Advanced Rider Assistance Systems (ARAS) Questionnaire	[69]	1
Likert Questionnaire	[70]	2
Interviewer-Administered Questionnaire	[71]	1
Malaysian School Zone Speed Limit (SZSL) Questionnaire Speed Limit (SZSL)	[72]	1
Indonesian Motorcycle Behaviour (Imrbq)	[73]	1

**Table 3:** Type of analysis for real-time studies.

Reference	Type of Analysis	Data Type (Collected by Author or NOT)	Number of Features Used (Speed, Time, Position, Turn Right, Turn Left, U-Turn, Zig-Zag, Sleepy, Deceleration, Acceleration, the Steering Angle and Steering Torque	Speed Feature	Position Feature	(Turn Right, Turn Left, U-Turn, Zig-Zag) Feature	Sleepy Feature	Deceleration Feature	Acceleration Feature	The Steering Angle and Steering Torque Feature	Average Age Factor	Gender Factor	The Selection Method of Most Effective Feature (RMSE, Algorithm, Het Map/Correlation Map	AI Algorithm Used	Type of Classification (Binary or, Multiclass)	Labelling Method	Type of Study (Small Scale or Large Scale)
[113]	Regression	by author	8	1	NA	1	1	1	1	NA	NA	NA	correlation map	ANN, SVM	multiclass	Manual	Small Scale (5 Rider)
[112]	NA	by author	4	NA	NA	NA	1	NA	1	1	NA	NA	NA	NA	NA	NA	Small Scale (5 Rider)
[110]	Statistical	by author	4	1	1	NA	NA	NA	NA	1	NA	Men	correlation map	(SVM)	multiclass	Manual (Pre-post) Experiment	Small Scale (12 Rider)
[108]	Descriptive Statistics	by author	1	1	NA	NA	NA	NA	NA	NA	25.6 years	NA	correlation map	NA	NA	NA	Small Scale (29 Rider)
[114]	NA	by author	5	NA	NA	1	NA	1	NA	NA	39 years	NA	random	(SVM), (HMMs),	multiclass	Automated (proprietary software BinAscii)	Small Scale (5 Rider)
[117]	statistical	by author	4	1	NA	NA	NA	NA	1	1	NA	NA	random	NA	NA	NA	Large Scale (7 Rider)
[111]	Descriptive statistic	author	4	1	NA	NA	NA	1	1	NA	NA	NA	from the literature review	NA	NA	NA	Small Scale (8 Rider)
[116]	NA	collected by author	3	1	NA	NA	NA	1	1	NA	NA	NA	NA	DBN, NB, SVM, and J48	Binary	Manual (Calculated Threshold)	Na

SVM = Support Vector Machine; NB = Naïve Bayes; NA = Not Available.

**Table 4:** Conditions of real-time experiments.

Ref	Country	Year	Road Type	Road Length	Time of Study	Weather Type	Traffic Type
[112]	Italy	2011	NA	NA	NA	NA	NA
[110]	Japan	2015	Cycle Sports Center	5km	NA	NA	NA
[114]	Australia	2015	urban areas (city)	2.5 km	day	sunny, rainy, and foggy	Variety of traffic conditions
[111]	India	2017	Main Road	14 km	peak hour on evening	NA	NA
[116]	Spain	2017	NA	NA	NA	NA	heavy traffic
[108]	Malaysia	2018	Highway (exclusive motorcycle lane)	20km	NA	NA	NA
[113]	Indonesia	2020	Highway	20 km/h to 50 km/h in 100 s	NA	NA	traffic jams
[117]	Spain	2021	highway, urban	78 km	NA	dry conditions, rain	NA

NA: Not Available.

**Table 5:** Countries, with references.

Countries	References
Australia	[8,15,62,76,94,118,127,130,162,165,200,201,202,203,204]
Athens	[153,179]
Argentina	[198]
Serbia	[155,205]
Brazil	[74,160,166,184,199]
USA	[3,8,19,54,91,123,143,150,152,168,169,172,180,187,191,206,207,208,209,210,211]
Cambodia	[212]
China	[14,26,40,63,79,81,90,99,161,188,190,195,211,213,214,215,216,217,218,219,220,221,222]
Denmark	[223]
France	[38,60,65,114,121,128,131,173,185,224,225]
Germany	[6,159,226,227,228]
India	[16,49,51,68,80,95,98,111,140,164,181,229]
Indonesia	[32,33,92,119,176]
Italy	[10,53,127,133,146,151,154,230,231,232]
Japan	[27,110,129,167,197,233,234]
Korea	[157,170,235]
Maldives	[25]
Malaysia	[66,67,78,84,96,108,158,186,192,236,237]
Slovenia	[47,75]
Singapore	[238]
Spain	[39,50,120,144,174,196,239,240]
Thailand	[18,97,122,171,193]
Turkey	[88]
UK	[37,56,58,139,141,175]
Vietnam	[3,163,194]
Iran	[12,17,64,100,115,116,124,156,241]

**Table 6:** References of previous data sources.

Data Sources	References
Survey, questionnaires or interview	[8,10,12,15,16,17,18,26,27,32,33,38,39,40,47,49,50,53,56,58,60,64,66,68,75,76,124,152,153,164,176,186,194,210,219,236,241,242]
Medical centres	[25,89,92,156,159,161,181,184,187,189,197,198,204]
Reports	[16,96,151,177,179,188,207]
Experiment and observation	[3,14,42,51,54,78,84,90,91,95,98,100,114,115,116,118,120,121,123,127,129,131,139,143,154,157,160,163,173,190,193,199,235,237,238]

**Table 7:** References of type of analysis.

Type of Analysis	References	Total
Descriptive Statistics	[174,181]	2
Sensitivity Analysis	[76,153,222]	3
Empirical Analysis	[16,19,78,169]	4
Qualitative Analysis	[92]	1
Confirmatory Factor Analyses	[39,49]	2
Data Distribution Analyses	[8]	1
Path Analysis	[39]	1
In-Depth Analysis	[68,115]	2
Meta-Analysis	[37,161]	2
Automated Video-Based Analysis Techniques	[68]	1
Linear Regression Analysis	[115]	1
Reference Analyses	[37]	1
Includes Bayesian-Related Analysis	[161]	1
Vibration Analysis	[14,196]	2
Macro And Micro Analyses	[165]	1
A Cross-Sectional	[162]	1
Chi-Square Analyses	[63,160]	2
Binary Logistic Regression	[184]	1
Multiple Regression Analysis	[25,49,236]	3
Headway Analysis	[119]	1
Statistical Analysis	[177,198]	2
odds ratios	[161]	1

**Table 8:** Sensors used in real-time motorcycle driver behaviour analyses.

Ref	GPS	Number of Motorcycles	Magnetic Sensor on the Wheel	Distance Sensor	Detection Range of Distance Sensor	Steering Angle Sensor	Steering Torque Sensor	Wheel Speed Sensor	Throttle Position Sensor	Eye Tracker Sensor	Gyroscope	Can-Bus Data (OBD)	Camera	Accelerometer	Smartphone	Installed DAS	DAS Type	Special Modifications	Complexity	Cost-Efficiency	Reliability of DAS
[113]	NA	1	NA	NA	NA	NA	NA	NA	NA	NA	1	NA	NA	1	Xiaomi Redmi 4A	No	Smartphone	No	VL	H	VL
[112]	NA	1	Yes	NA	NA	Yes	Yes	NA	NA	NA	1	NA	1	3	NA	Yes	Camera on Back of Motorcycle + Plate on Driver Back	Yes	VH	VL	VH
[110]	1	1	NA	NA	NA	NA	NA	NA	NA	Field + Movement Cameras	2	NA	1	NA	NA	No	An instrument-equipped helmet	No	M	L	H
[108]	1	1	NA	Yes	5 cm–3 m	NA	NA	NA	NA	NA	NA	1	2	1	NA	No	Data Logger (speed + Video) + Arduino + Range Sensor	No	H	L	M
[114]	NA	1	NA	NA	NA	Yes	NA	Yes	Yes	NA	1	NA	4	1	NA	YES	Embedded Datalogger [Video Logger]	Yes	VH	VL	VH
[117]	1	3 + 1 Backup	NA	NA	NA	NA	NA	NA	NA	1	NA	1	2	NA	NA	Yes	CAN-BUS datalogger + Camera on Helmet	No	H	L	H
[111]	1	Many	NA	NA	NA	NA	NA	NA	NA	NA	NA	1	NA	NA	NA	No	OBD data logger	No	M	VH	VL
[116]	1	1	NA	NA	NA	NA	NA	NA	NA	NA	1	NA	NA	1	Yes	No	Simple Smartphone + Wristband for Health Monitoring	No	M	H	L

NA: Not Available; VH: Very High; H: High; M: Medium; L: Low; VL: Very Low.

**Table 9:** Present comparison between our works versus review articles.

Ref	Year	Area	Type	Type of Factor	Taxonomy	AI Models Analysis	DAS and Sensors
[163]	2016	Safety	Review	Cooperative driving	NA	No	No
[150]	2018	Traffic and safety	Critical review	Behaviour	NA	No	Yes
[3]	2018	Safety of PTWs	Review	PTW driver behaviour and attitudes	NA	No	Yes
[149]	2018	Traffic and safety	SLR	Human factors	NA	No	No
[245]	2019	Traffic and safety	SLR	Behaviour	NA	No	No

PTWs: powered two-wheelers; SLR: a systematic review; NA: Not Available; AI: Artificial Intelligence.

## Data Availability

Not applicable.
